# Network meta-analysis for an ordinal outcome when outcome categorization varies across trials

**DOI:** 10.1186/s13643-024-02537-w

**Published:** 2024-05-09

**Authors:** Paul Morris, Chong Wang, Annette O’Connor

**Affiliations:** 1 Department of Statistics, Iowa State University, Ames, 50010 IA USA; 2 Department of Veterinary Diagnostic and Production Animal Medicine, Iowa State University, Ames, 50011 IA USA; 3 Department of Large Animal Clinical Sciences, Michigan State University, East Lansing, 48824 MI USA

**Keywords:** Network meta-analysis, Incomplete data, Evidence synthesis

## Abstract

**Background:**

Binary outcomes are likely the most common in randomized controlled trials, but ordinal outcomes can also be of interest. For example, rather than simply collecting data on diseased versus healthy study subjects, investigators may collect information on the severity of disease, with no disease, mild, moderate, and severe disease as possible levels of the outcome. While some investigators may be interested in all levels of the ordinal variable, others may combine levels that are not of particular interest. Therefore, when research synthesizers subsequently conduct a network meta-analysis on a network of trials for which an ordinal outcome was measured, they may encounter a network in which outcome categorization varies across trials.

**Methods:**

The standard method for network meta-analysis for an ordinal outcome based on a multinomial generalized linear model is not designed to accommodate the multiple outcome categorizations that might occur across trials. In this paper, we propose a network meta-analysis model for an ordinal outcome that allows for multiple categorizations. The proposed model incorporates the partial information provided by trials that combine levels through modification of the multinomial likelihoods of the affected arms, allowing for all available data to be considered in estimation of the comparative effect parameters. A Bayesian fixed effect model is used throughout, where the ordinality of the outcome is accounted for through the use of the adjacent-categories logit link.

**Results:**

We illustrate the method by analyzing a real network of trials on the use of antibiotics aimed at preventing liver abscesses in beef cattle and explore properties of the estimates of the comparative effect parameters through simulation. We find that even with the categorization of the levels varying across trials, the magnitudes of the biases are relatively small and that under a large sample size, the root mean square errors become small as well.

**Conclusions:**

Our proposed method to conduct a network meta-analysis for an ordinal outcome when the categorization of the outcome varies across trials, which utilizes the adjacent-categories logit link, performs well in estimation. Because the method considers all available data in a single estimation, it will be particularly useful to research synthesizers when the network of interest has only a limited number of trials for each categorization of the outcome.

## Background

Network meta-analysis (NMA) is an extension of traditional pairwise meta-analysis that allows for the simultaneous comparison of multiple interventions by utilizing direct and indirect evidence from a network of randomized clinical trials [1]. When the outcome of interest is categorical with more than two categories, the NMA is typically conducted through the use of a generalized linear model (GLM) where the random component is multinomial, as described by [2]. It is common to utilize some type of logit link in a multinomial GLM [3], under which the parameters of interest in the NMA correspond to log-odds ratios of subjects belonging to a given category versus another under a particular intervention relative to the network’s baseline intervention. The type of logit employed determines the outcome categories under consideration in these comparative effect parameters. When the outcome is unordered, the baseline-category logit is often used [4], and the log-odds ratios are parameterized in terms of non-baseline categories versus a selected baseline category. When the outcome is ordered, it is often referred to as an ordinal variable with the categories referred to as levels. One possible choice of link function for analyzing an ordinal outcome is the adjacent-categories logit [3], under which the log-odds ratios are parameterized in terms of adjoining levels. Analysts can select the logit link that best matches their question of interest given the properties of the outcome.

While ordinal outcomes must be comprised of mutually exclusive and exhaustive levels, reporting of data for such an outcome can depend on the question of interest being addressed in a given trial. This often leads to networks for which the categorization of the outcome varies across trials. For example, suppose that we are interested in the effects of a set of interventions on an ordinal outcome with four mutually exclusive and exhaustive levels, call them A, B, C, and D. While some trials report event counts for each of the four levels, others may report combined values for B and C or even for B, C, and D. Data combined in this fashion has been referred to as incomplete [5]. Note that incomplete data in the sense presented here is unique to categorical outcomes and is distinct from the phenomenon of missing data for which the values for some subjects are either not measured or not reported at all [6–8]. Trials that report incomplete data can still provide information that contributes to our knowledge of the underlying comparative effects. For example, if we are utilizing the adjacent-categories logit link, the combined data for levels B and C can inform the estimates of the log-odds ratios involving B versus A and D versus C. To maximize the utility of the network of trials, it would be advantageous to consider all of the available data in estimation, regardless of outcome categorization. Unfortunately, the standard multinomial GLM framework cannot simultaneously incorporate data from multiple categorizations without some modification.

The problem of multiple outcome categorizations within a network has been addressed by [5] for the case of an unordered outcome. They proposed an extension to the multinomial GLM framework wherein the form of the multinomial likelihood was modified to allow for outcome categories to be combined according to a trial’s categorization. Their model incorporates random comparative effects and utilizes the baseline-category logit link. However, the modified multinomial likelihood was structured around a specific example and is therefore not provided in a general form. In addition, the authors did not evaluate the performance of the model through simulation.

In this paper, we extend the method developed by [5] on several fronts. First, we take a step toward adapting the method for the case of an ordinal outcome by proposing a model that utilizes the adjacent-categories logit link rather than the baseline-category logit. The structure of the adjacent-categories logit link takes into account the ordering of the outcome [3] and is particularly useful if we are interested in the log-odds ratios pertaining to adjoining levels, as parameters representing these comparisons are directly included in the model. Second, we provide the general form of the modified multinomial likelihood that allows for any outcome categorization, whether the outcome is ordered or unordered. Throughout, we assume that the intervention effects are fixed, although this can be extended to the random effects case in a manner similar to that presented in [5], and use a Bayesian approach by conducting estimation through Markov Chain Monte Carlo (MCMC).

The remainder of the paper is organized as follows. The “Methods” section details the proposed model, including the general form of the modified multinomial likelihood, and describes the approach to estimation. The “Application” section illustrates a use case of the proposed model through an analysis of a real network of trials comparing the effects of various regimens of antibiotics on the prevention of liver abscesses in beef cattle, where the severity of the abscesses is reported on an ordinal scale. The “Simulation” section presents a simulation study that assesses the estimation performance of the proposed model, and the “Discussion” and “Conclusions” sections discuss and conclude.

## Methods

This section specifies the proposed NMA model for an ordinal outcome when outcome categorization varies across trials. This includes detailing the general form of the modified multinomial likelihood and the model for the response probabilities that follows from use of the adjacent-categories logit link. The proposed model is a modified version of that detailed in [5] for an unordered outcome, with some of the notation borrowed from [2]. The Bayesian estimation approach used throughout, including the specification of priors and starting values, is also presented.

### Accommodating multiple outcome categorizations

Here the general form of the modified multinomial likelihood that allows for any outcome categorization is specified. While the term levels will be used to denote the outcome categories, this notation is also applicable to the unordered case. Consider *K* interventions compared in *I* trials, where trial *i* has $n_i$ arms. Suppose that the outcome consists of *M* mutually exclusive and exhaustive levels, but that a given trial need not report data for each level separately. Rather, data for some levels may be reported together. That is, suppose that trial *i* collapses the *M* levels into $M_i \le M$ groups denoted by $A_{i,1}, A_{i,2}, \ldots , A_{i,M_i}$ that are mutually exclusive and exhaustive. Let $\left( r_{i,k,1},\ldots ,r_{i,k,M}\right)$ be the vector of true, but potentially unreported, counts for the *M* levels under trial *i* and intervention *k*. We can denote the total count for the $c^{th}$ combined category under trial *i* and intervention *k* as

1 $$\begin{aligned} z_{i,k,c} = \sum \limits _{m \in A_{i,c}}r_{i,k,m}, \end{aligned}$$

where the separate counts $r_{i,k,m}$ for $m \in A_{i,c}$ are unreported if they belong to a group of combined levels. This notation for the grouping of levels is adapted from that given in [9] for the form of the collapsed and partitioned multinomial distribution. Note that this notation is applicable to both unordered and ordinal outcomes, but under the ordinal case it is reasonable to restrict groups to include only adjacent categories.

The reported data for intervention *k* in study *i* can then be modeled using a multinomial likelihood:

2 $$\begin{aligned} L(\varvec{p}_{i,k}|{\textbf {z}}_{i,k}) = \frac{N_{i,k}!}{z_{i,k,1}!\dots z_{i,k,M_i}!}\left( \sum \limits _{m \in A_{i,1}}p_{i,k,m}\right) ^{z_{i,k,1}} \dots \left( \sum \limits _{m \in A_{i,M_i}}p_{i,k,m}\right) ^{z_{i,k,M_i}}, \end{aligned}$$

where $\varvec{p}_{i,k}$ and ${\textbf {z}}_{i,k}$ are the response probability and observed count vectors for intervention *k* in trial *i*, $p_{i,k,m}$ is the true response probability for intervention *k* and level *m* in study *i*, and $N_{i,k} = \sum _{c=1}^{M_i}z_{i,k,c} = \sum _{m=1}^{M}r_{i,k,m}$. Since the levels are mutually exclusive and exhaustive, $\sum _{c=1}^{M_i}\sum _{m \in A_{i,c}}p_{i,k,m} = \sum _{m=1}^{M}p_{i,k,m} = 1$ for each trial *i* and intervention *k*.

Following independence, the likelihood for the entire network is then

3 $$\begin{aligned} L(\varvec{p}|{\textbf {z}}){} & {} = \prod _{i = 1}^{I}\prod _{k\in K_{i}}L(\varvec{p_{i,k}|{\textbf {z}}_{i,k}}) \nonumber \\{} & {} = \prod _{i = 1}^{I}\prod _{k\in K_{i}}\frac{N_{i,k}!}{z_{i,k,1}!\dots z_{i,k,M_i}!}\left( \sum \limits _{m \in A_{i,1}}p_{i,k,m}\right) ^{z_{i,k,1}} \dots \left( \sum \limits _{m \in A_{i,M_i}}p_{i,k,m}\right) ^{z_{i,k,M_i}}, \end{aligned}$$

where $\varvec{p}$ and ${\textbf {z}}$ are the response probability and observed count vectors for the entire network and $K_{i}$ denotes the group of $n_i$ interventions included in trial *i*.

The modified likelihood accommodates the reporting of data for combined levels through the incorporation of the total outcome counts and response probabilities of the respective levels. The trials that combine levels therefore provide partial information on the underlying response probabilities [5], allowing for all data available across the network to contribute to the estimation of the model parameters.

### Model for the response probabilities

When working with an ordinal outcome, it is natural to be interested in comparisons of interventions that involve the underlying order. A link function should be selected such that it allows analysts to directly make those comparisons of interest. The cumulative logit and the adjacent-categories logit links are common choices for an ordinal outcome. However, if proportional odds are not assumed, use of the cumulative logit does not necessarily result in valid estimated response probabilities [3]. In the context of NMA, proportional odds implies that the comparative effects of interventions are identical for each of the level pairings considered under the chosen link function. The proportional odds assumption can be useful, particularly if there is reason to believe that the effects of interventions are similar across each of the level pairings, because it utilizes the ordinality of the outcome to reduce the number of parameters included in the model [3]. We leave the development of a procedure for determining when it is appropriate to assume proportional odds under the given framework for future work, and opt to utilize the adjacent-categories logit link under non-proportional odds for the remainder of this paper. Under the adjacent-categories logit link, the comparative effects are log-odds ratios of a subject belonging to a given level relative to the level below it. This could be of interest, for example, if we have a network where the outcome consists of the severity of disease ranging from healthy to severe. The estimates of the comparative effects would then provide insight on how interventions affect the odds of a subject belonging to the mild relative to the healthy state, the moderate relative to the mild state, and the severe relative to the moderate state. Since the adjacent-categories logit and baseline-category logit are functionally reparameterizations of each other [3], such comparisons could be made under the baseline-category logit link indirectly. However, the use of the adjacent-categories logit puts parameters corresponding to the comparisons of interest directly in the model. This approach can be helpful when specifying priors, making inferences, and diagnosing problems with estimation.

Since each trial does not necessarily include each of the *K* interventions, some additional notation is necessary. An overall baseline intervention for the network, *b*, must be selected. This will often correspond to a placebo or standard therapy group. Each trial *i* also has a trial-specific baseline intervention, $b_i$, which will be the same as *b* if $b \in K_i$. Then the response probabilities can be modeled as

4 $$\begin{aligned} \theta _{i,k,m} = log\left( p_{i,k,m}/p_{i,k,m-1}\right) = \mu _{i,m} + d_{b_i,k,m}I(k \ne b_i) \quad \text {for } m = 2,\ldots ,M, \end{aligned}$$

where $I(\cdot )$ denotes the indicator function, and

5 $$\begin{aligned} \theta _{i,k,m} = 0 \quad \text {for } m = 1. \end{aligned}$$

The logit described in Eq. 4 can be interpreted as the log-odds of a subject belonging to level *m* versus $m-1$ under trial *i* and intervention *k*. Under Eq. 4, the $\mu _{i,m}$ correspond to trial-specific baselines representing the log-odds of level *m* versus level $m-1$ under study *i*’s baseline intervention $b_i$. These are regarded as nuisance parameters and serve only to set up the contrast needed to include the parameters corresponding to the comparative effects. The $d_{b_i,k,m}$ represents the log-odds ratio of a subject belonging to category *m* versus $m-1$ under intervention *k* relative to the trial-specific baseline $b_i$. These are included in Eq. 4 only if $k \ne b_i$, as if $k = b_i$ then $\mu _{i,m}$ represents the corresponding logit on its own. Following from the consistency assumption (see [10] for an overview of the assumptions commonly made in NMA),

6 $$\begin{aligned} d_{b_i,k,m} =\left\{ \begin{array}{ll} d_{k,m} - d_{b_i,m} &{}b_i \ne b\\ d_{k,m} &{}b_i = b \end{array}\right. \end{aligned}$$

where $d_{k,m}$ represents the log-odds ratio of a subject belonging to category *m* versus $m-1$ under intervention *k* relative to the overall baseline intervention *b*. The $d_{k,m}$ are the parameters of interest and do not vary across trials under the assumption of fixed intervention effects. Under the consistency assumption, the comparative effect between any two interventions $k_1$ and $k_2$ is $d_{k_1, k_2, m} = d_{k_2,m} - d_{k_1,m}$.

Expressions for the response probabilities can be obtained through an application of the inverse adjacent-categories logit function [11] to Eqs. 4 and 5:

7 $$\begin{aligned} p_{i,k,m} = exp\left( \sum \limits _{l=1}^{m}\theta _{i,k,l}\right) /\left( 1 + \sum \limits _{m = 2}^{M}\exp \left( \sum \limits _{l=1}^{m}\theta _{i,k,l}\right) \right) \end{aligned}$$

and

8 $$\begin{aligned} p_{i,k,1} = 1 - \sum \limits _{m=2}^{M}\pi _{i,k,m}. \end{aligned}$$

### Bayesian implementation

The model parameters are estimated using MCMC via the JAGS software package [12]. The Bayesian approach is advantageous for the proposed method, as it allows for the specification of informative priors to help overcome the lack of identifiability of some of the trial-specific baseline parameters.

#### Prior specification

In order to implement the Bayesian approach, priors need to be assigned to the parameters in the model. Since we are assuming fixed intervention effects, we need only consider each of the $\mu _{i,m}$ and $d_{k,m}$. Identifiability of the $d_{k,m}$ requires that for each adjacent pair of levels there exists a path of comparisons that connects all of the interventions such that in each comparison the event counts for the two levels are reported separately. Assuming that this condition holds, we can assign these parameters non-informative $\text {Normal}(0, 1,000,000)$ priors as was done in [5].

Recall that the $\mu _{i,m}$ are trial-specific baselines representing the log-odds of a subject belonging to level *m* versus $m-1$ under trial *i*’s baseline intervention $b_i$. Since any combination of adjacent levels is allowed in the observed data, for a given trial, separate event counts for levels *m* and $m-1$ may not be reported. This means that there will not be data available to estimate some of the $\mu _{i,m}$ parameters. Stronger priors can be used to help overcome this lack of identifiability. We propose the following procedure to specify priors for the $\mu _{i,m}$. For the group of trials *T* that report separate event counts for levels *m* and $m-1$ under trial-specific baseline intervention $b_i$:

1. Calculate the empirical log-odds for category *m* versus $m-1$ under intervention $b_i$ from the available data. In the specified notation, these would take the form $y_i = log(r_{i,b_i,m}/r_{i,b_i,m-1})$ for trials $i \in T$. Note that if either of the event counts is 0 for trial *i*, we add 0.5 to each count to ensure that the empirical log-odds fall on the real line.
2. Fit the following Bayesian model using the $y_i$ from the previous step as data: $y_i {\mathop {\sim }\limits ^{iid}} \text {Normal}(\mu , \sigma ^2)$ with priors $\mu \sim Normal(0, 1000)$ and $\sigma \sim Uniform(0, 5)$. This can be done using JAGS via the *rjags* R package [13], where 10,000 iterations are run for each of burn-in and sampling. Note that this step is adapted from the estimation of the baseline-effects model under NMA for a binary outcome presented in [14].
3. Letting $\hat{\mu }$ and $\hat{\sigma }^2$ denote the posterior means of $\mu$ and $\sigma ^2$ obtained in step 2, assign the prior $\mu _{i,m} \sim Normal\left(\hat{\mu }, \hat{\sigma }^2\right)$ for each $i \in T$.

#### Selection of starting values

Selecting suitable starting values is important to ensure proper behavior of the MCMC chains. Schmid et al. [5] proposes a method for selecting dispersed starting values under the baseline-category logit link. This procedure was later implemented in the *BNMA* R package [15]. We modify this procedure for use with the adjacent-categories logit link by substituting the empirical adjacent-categories log-odds for the empirical baseline-category log-odds in the described regression.

#### Parameter estimation

Estimation was achieved through MCMC using the JAGS software, where we interfaced with JAGS via the *rjags* R package. Four MCMC chains were used, and proper convergence and mixing of the chains were monitored through the Gelman-Rubin diagnostic [16] and examination of the trace plots.

## Application

In this section, the use of the proposed model is illustrated through the analysis of a network of studies examining the effects of several antibiotic regimens on the prevention of liver abscesses in beef cattle. Many studies in the veterinary literature have compared the efficacy of various interventions on this outcome, but synthesis of this research has proved difficult because the categorization of the outcome varies across trials. Abscess severity is often measured using an ordinal scale containing four levels: healthy (H), one or two small abscesses (A−), two to four small abscesses (A), and one or more large abscesses (A+) [17, 18]. This ordinal scale is well established in the beef cattle industry. However, because the economic impact of liver abscesses is mainly linked to the A+ level, some investigators combined levels A− and A [19, 20], resulting in a three-level outcome variable. This three-level scale is currently used by the well-known Elanco Liver Check Service [21]. Still other investigators report the presence of any abscess regardless of severity, combining A−, A, and A+ [22, 23], resulting in a two-level outcome variable. To control liver abscesses, in-feed antibiotics are used. Currently, in-feed tylosin phosphate, an antibiotic in the same family as erythromycin, is the primary approach to the prevention of liver abscesses. However, over the years numerous approaches to control have been evaluated including diets, other antibiotics, non-antibiotic additives, ionophores, and other regimens of tylosin.

The data for this network are a subset of that obtained from a systematic review of interventions aimed at preventing liver abscesses in cattle (the review protocol of which is available at https://syreaf.org/protocols/). For the purposes of this project, only four interventions are included in the network presented here to enable focus on the methodological issue of interest. Three of the intervention groups are regimens of the antibiotics tylosin phosphate or virginiamycin while the fourth is a placebo group. An example of a trial identified by the review but excluded in this study is [24]. The purpose of this trial was to evaluate the effect of a phytogenic feed additive (Digestarom; Biomin, Getzersdorf, Austria) on multiple outcomes including liver abscesses in finishing steers. Since the trial did not examine any of the tylosin phosphate or virginiamycin regimens of interest it was excluded from our network.

For the trials included in the network, we defined a placebo arm as any trial arm that did not contain tylosin phosphate or virginiamycin. Placebo arms contained any level of monensin or diet composition. Monensin is an ionophore administered in feed that promotes the efficient use of feedstuffs and is not considered to have any impact on liver abscesses. As many trials included multiple such arms, the data from these arms were combined to create a single placebo arm per trial. To illustrate this approach we use a trial published in [17]. This trial was a 2 by 3 factorial design with one factor being diet: based on steam-flaked corn finishing diet (SFC) or SFC plus 25% (dry basis) corn wet distillers grains with solubles (WDGS). The second factor was feed additives: no added antibiotics (NONE), 300 mg of monensin daily (MON), or 300 mg of monensin + 90 mg of tylosin phosphate daily (MON+TYL). Our approach to handling such a trial was to combine the data for the NONE + SFC, NONE + WDGS , MON + SFC, and MON + WDG arms into a single placebo arm. The antibiotic arm (tylosin phosphate) was created by combing the data from the (MON + TYL) + SFC and (MON + TYL) + WDGS arms. The treatments were fed from arrival to slaughter, i.e., 150 days.

Tylosin phosphate arms were categorized based on dosing regimens as follows:

- Protocols that began the feeding period without tylosin phosphate and started to feed constantly late in the feed period (latestart)
- Protocols that began the feeding period with tylosin phosphate and ended the feeding period without tylosin phosphate (earlyfinish)
- Protocols that did use tylosin phosphate for the entire feeding period but limited the period to less than or equal to 100 days (short)
- Protocols that did use tylosin phosphate for the entire feeding period but that feeding period was more than 100 days (long)
- Protocols that did use tylosin phosphate for the entire period but intermittently (intermittent).

An example of the use of this approach to categorize tylosin regimens is provided by [25], which investigated management strategies that reduce in-feed tylosin phosphate in the control of liver abscesses in feedlot cattle. A total of 7576 crossbred yearlings were allocated to the trial (approximately 253 animals/pen with 10 replicate pens per treatment) and individually randomized to one of three treatments: tylosin phosphate (11 ppm) was included in-feed (1) for the first 125 days on feed (DOF) (earlyfinish) (2) for DOF 41 to 161 (latestart) or (3) for the entire feeding period for DOF 0 to 161 (long). However, for this project, we only included arms corresponding to the “long” regimen as a means of keeping the illustrative data set simple. This is also the registered dose, while the others are exploratory.

Virginiamycin arms were categorized based on two dose levels: less than 15 mg/kg, and greater than or equal to 15 mg/kg. An example of an application of this grouping scheme follows from [26], which fed cattle a four-level range of virginiamycin (0, 10, 25, and 50 mg/kg) over multiple trials throughout a 245 day growing-finishing period. For this trial, the 0 mg/kg arm was designated as the placebo, 10 mg/kg arm was designated as being less than 15 mg/kg, and the data for the 25 mg/kg and 50 mg/kg arms were combined into a single arm with greater than 15 mg/kg virginiamycin.

**Fig. 1:**
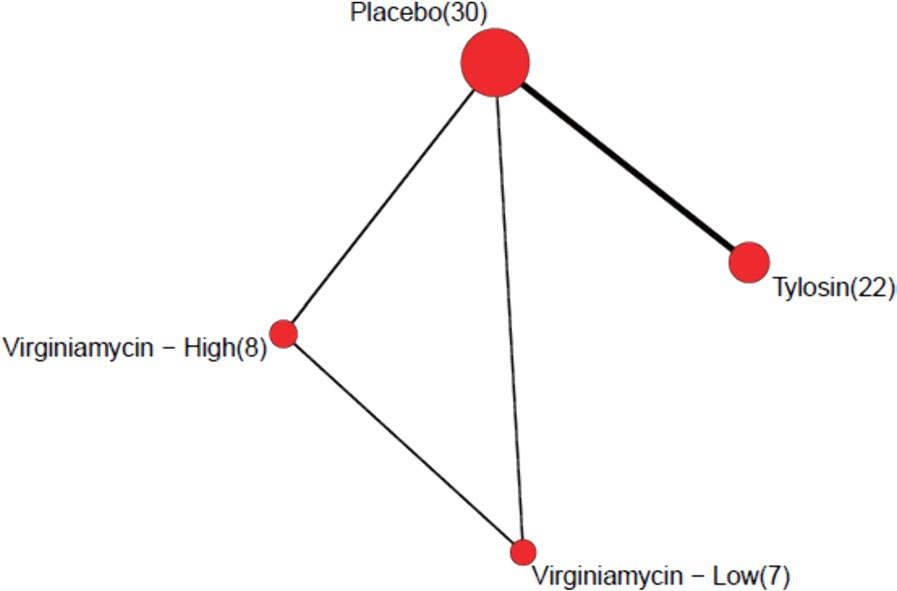
Diagram of the liver abscess trial network. Nodes are interventions and edges are direct comparisons. The size of the nodes and the numbers in parentheses indicate the number of trials that include each intervention, while the edge width indicates the number of direct comparisons made between each intervention

Figure 1 presents a diagram of the network. Note that every trial included a placebo group, and that the tylosin phosphate regimen is included in a large number of trials relative to the virginiamycin regimens. Also note that the tylosin phosphate regimen is not directly compared with either of the virginiamycin dosing regimens. Each of the three liver abscess categorizations detailed in the first paragraph of this section is present in the network. Table 1 details the frequencies of these categorizations. While 12 of the 22 trials comparing tylosin phosphate to the placebo reported complete data (4 levels), complete data was available in only four of the eight trials including either of the virginiamycin regimens. The remaining four trials that included either of the virginiamycin regimens combined A−, A, and A+ in their reporting. There is thus substantially less data available to estimate the comparative effects involving virginiamycin compared to those for tylosin phosphate.

**Table 1:** Frequencies of each of the outcome categorizations. The second column contains the total number of trials utilizing each reporting pattern while the other columns contain the total number of arms for each intervention that belong to those trials

Reporting pattern	Trials	Placebo	Tylosin	Virginiamycin - low	Virginiamycin - high
H, (A− and A), A+	4	4	4	0	0
H, A−, A, A+	16	16	12	4	4
H, (A− and A and A+)	10	10	6	3	4

Estimation was conducted as described in the “Bayesian implementation” section. Here the model parameters were estimated using 50,000 iterations for each of burn-in and sampling. Table 2 displays the point estimates and 95% credible intervals for each of the basic comparative effect parameters on the log-odds ratio and odds ratio scales. A negative estimate on the log-odds ratio scale means that a subject is estimated to be relatively more likely to belong to the lower disease level than the higher one under the noted intervention compared to under the placebo. We note that the 95% credible intervals are quite wide for the comparative effects associated with virginiamycin due to the limited amount of data on that antibiotic in the network.

**Table 2:** Estimates of the$d_{k,m}$on the log-odds ratio (LOR) scale. Interventions are compared to placebo. Point estimates are posterior means and central 95% credible intervals are also reported. Estimates and credible intervals are also presented on the odds ratio (OR) scale to assist with interpretation

Intervention	Level	Estimate (LOR)	95% CI (LOR)	Estimate (OR)	95% CI (OR)
Tylosin - long	A− to H	− 0.850	(− 1.022, − 0.682)	0.427	(0.360, 0.506)
Tylosin - long	A to A−	− 0.147	(− 0.416, 0.121)	0.863	(0.660, 1.129)
Tylosin - long	A+ to A	− 0.296	(− 0.543, − 0.041)	0.744	(0.581, 0.959)
Virginiamycin - low	A− to H	0.001	(− 0.364, 0.360)	1.001	(0.695, 1.434)
Virginiamycin - low	A to A−	− 0.305	(− 0.918, 0.295)	0.737	(0.399, 1.343)
Virginiamycin - low	A+ to A	0.351	(− 0.183, 0.891)	1.420	(0.833, 2.437)
Virginiamycin - high	A− to H	− 0.511	(− 0.840, − 0.162)	0.600	(0.432, 0.850)
Virginiamycin - high	A to A−	0.077	(− 0.470, 0.613)	1.080	(0.625, 1.845)
Virginiamycin - high	A+ to A	− 0.334	(− 0.814, 0.146)	0.716	(0.443, 1.157)

## Simulation

In this section, properties of the estimates of the comparative effect parameters are evaluated through simulation under two scenarios. In the first, we treat the point estimates obtained in the “Application” section as the true values for the parameters and use these values to repeatedly regenerate the data for the liver abscess network. The estimates obtained by analyzing the regenerated datasets can then be used to calculate bias, root mean square error (RMSE), and coverage probability of the credible intervals. The second scenario is similar to the first, except that here in the regeneration step data for each study is generated ten times as if it came from ten different studies. This results in simulated datasets that are ten times larger than those in the first scenario, which allows for the evaluation of large-sample estimation performance.

### Scenario I

The simulation procedure for the first scenario is as follows:

1. Obtain the posterior means for each parameter from the existing NMA (values for the comparative effects are given in Table 2, while those for the trial-specific baselines are not shown). For each trial *i*, intervention *k* and outcome level $m = 2,\ldots , M$ that occurs in the network, let $\hat{\mu }_{i,m}$ and $\hat{d}_{k,m}$ denote the corresponding posterior means.
2. For each trial *i*, intervention *k* and outcome level $m = 2,\ldots , M$ in the network, calculate $\hat{\theta }_{i,k,m} = \hat{\mu }_{i,m} + \hat{d}_{b_i,k,m}$ where $\hat{d}_{b_i,k,m}$ is obtained by using the estimated comparative effects in Eq. 6, i.e., the consistency assumption. Then obtain values for the multinomial response probabilities, denoted by $\hat{p}_{i,k,m}$, using $\hat{\theta }_{i,k,m}$ in Eqs. 7 and 8.
3. Complete the following 1000 times:
  - Generate the complete data for the network using the calculated probabilities. For each trial *i* and intervention *k* in $K_i$: $(r_{i,k,1},\ldots ,r_{i,k,M}) \sim \text {Multinomial}(N_{i,k}, (\hat{p}_{i,k,1},\ldots ,\hat{p}_{i,k,M}))$.
  - Combine the outcome data where necessary as indicated by the existing network. This gives the observed data in the form of the $z_{i,k,c}$, where $z_{i,k,c} = \sum _{m \in A_{i,c}}r_{i,k,m}$ and $A_{i,c}$ is the $c^{th}$ group of outcome levels for trial *i*.
  - Conduct the analysis on the generated dataset using the proposed model as was done in the “Application” section. Here 25,000 iterations were used for each of burn-in and sampling to ensure reasonable computation time. Record the posterior means of the $d_{k,m}$, the comparative effect parameters of interest.
4. Calculate the bias, RMSE, and coverage probability for each of the $d_{k,m}$ using the posterior means as the point estimates.

The simulation results for the first scenario are displayed in Table 3. We see that the magnitude of the biases is relatively small and consistent across each of the comparative effect parameters. Given the lack of available data with which to estimate the trial-specific baseline parameters in some trials, some bias is expected as posterior draws for the baseline parameters that fall far from the true values will inevitably affect the estimation of the comparative effects. The use of more informative priors for the trial-specific baseline parameters as described in the “Prior specification” section helps limit the bias relative to using non-informative priors. The RMSEs are somewhat large due to the limited amount of data available to estimate many of these comparisons, particularly for those involving either of the virginiamycin regimens. As we will see in the simulation results for the second scenario, the magnitude of the RMSEs can be reduced if more data is available. Finally, it is clear that the proposed model achieves coverage probabilities close to the nominal value.

**Table 3:** Simulation results for scenario I (30 trials). Properties of estimates of the basic comparative effect parameters on the log-odds ratio scale. Interventions are compared to placebo

Intervention	Level	True value	Bias	RMSE	Coverage probability
Tylosin - long	A− to H	− 0.850	− 0.013	0.085	0.946
Tylosin - long	A to A−	− 0.147	− 0.017	0.134	0.950
Tylosin - long	A+ to A	− 0.296	0.012	0.125	0.951
Virginiamycin - low	A− to H	0.001	− 0.028	0.178	0.953
Virginiamycin - low	A to A−	− 0.305	0.024	0.315	0.948
Virginiamycin - low	A+ to A	0.351	− 0.010	0.288	0.950
Virginiamycin - high	A− to H	− 0.511	− 0.031	0.161	0.951
Virginiamycin - high	A to A−	0.077	0.046	0.269	0.949
Virginiamycin - high	A+ to A	− 0.334	− 0.028	0.247	0.950

### Scenario II

For the second scenario, the simulation procedure is identical to that used in the first except that in step (3), each trial in the existing network is used to generate data for ten separate trials rather than one. We thus generate datasets that are ten times as large as those under the first scenario. Results of the simulations under the second scenario are available in Table 4. Note that the biases are of similar magnitude to those seen under the first scenario. While the datasets are ten times as large, there are also ten times as many trials, and thus we have not circumvented the issue brought on by the trial-specific baseline parameters that was noted in the first scenario. However, the magnitude of the RMSEs is substantially reduced, including those corresponding to comparative effects involving the virginiamycin interventions. It is worth noting here that the coverage probabilities are slightly lower than in the previous scenario. Since more data is available to estimate each of these comparisons, the credible intervals are narrower. In conjunction with the slight bias introduced through the trial-specific baseline parameters, the narrower intervals result in the true values falling outside of the given bounds at a higher rate.

**Table 4:** Simulation results for scenario II (300 trials). Properties of estimates of comparative effect parameters on the log-odds ratio scale. Interventions compared to placebo

Intervention	Level	True value	Bias	RMSE	Coverage probability
Tylosin - long	A− to H	− 0.850	− 0.008	0.027	0.943
Tylosin - long	A to A−	− 0.147	− 0.020	0.047	0.925
Tylosin - long	A+ to A	− 0.296	0.011	0.041	0.940
Virginiamycin - low	A− to H	0.001	− 0.020	0.059	0.936
Virginiamycin - low	A to A−	− 0.305	0.035	0.103	0.934
Virginiamycin - low	A+ to A	0.351	− 0.027	0.093	0.940
Virginiamycin - high	A− to H	− 0.511	− 0.027	0.056	0.922
Virginiamycin - high	A to A−	0.077	0.046	0.094	0.918
Virginiamycin - high	A+ to A	− 0.334	− 0.027	0.081	0.938

## Discussion

In this paper we proposed a fixed effect multinomial NMA model for an ordinal outcome that allows for multiple outcome categorizations within a network. The proposed model is a modification of that presented in [5] for an unordered categorical outcome. These models are particularly useful when working with sparse networks, which are commonly encountered and can affect the quality of comparative effect estimates in terms of both precision and power. Because the models simultaneously accommodate trials with different outcome categorizations, they allow for the consideration of all available data in the estimation of the comparative effects. Practitioners can therefore avoid contributing to the sparsity of the network by excluding valuable information.

Furthermore, when working with an ordinal outcome, it is often desirable to compare interventions in such a way that the ordering is recognized. While it would be possible to use the method of [5] to analyze a network with an ordinal outcome, the resulting comparative effects would not directly recognize the ordinality. The adjacent-categories logit link incorporates the ordering into the comparative effect parameters and is an appropriate choice in many applications. It is possible to back out the adjacent-category comparative effect estimates from those of the baseline-category model through the relationship between the two logit functions [3], but including the adjacent-category comparative effects directly in the model makes specifying priors, conducting inference, and optimizing estimation more straightforward.

As with any method, there are some limitations that are important to keep in mind. While the simulations showed that the estimates of the comparative effects behave reasonably well, the lack of data with which to estimate some of the trial-specific baseline parameters introduces some bias that is not eliminated as sample size increases (see Tables 3 and 4). An empirical approach to specifying informative priors for these trial-specific baseline parameters was used in an attempt to keep the bias small. This approach limits the bias relative to using non-informative priors but could potentially be optimized even further. It is important to note that there is then a potential trade-off between limiting sparsity and introducing bias that comes with allowing for multiple outcome categorizations. It is possible that the standard multinomial NMA model could be the better choice for some networks with sizable amounts of data.

It is also important to ensure that the estimation procedure behaves as intended. Even with sensibly chosen priors and starting values, the complexity of NMA models can make estimation through MCMC difficult. For example, in the second simulation scenario, the adaptation phase run by JAGS was not completed for many of the generated datasets even after 25,000 iterations. Adaptation can affect the behavior of the samplers employed by JAGS. Care therefore needed to be taken to ensure that the chains were run long enough such that mixing and convergence were achieved and effective sample sizes were reasonably high.

Finally, additional developments not implemented here can increase the utility of the proposed method. For example, the proportional odds assumption, which states that the comparative effect parameters for a given intervention are the same across the different level pairings, could be reasonable for some applications with ordinal outcomes. The assumption exploits the ordinality of the outcome to reduce the number of parameters included in the model [3]. However, it is a strong assumption to make and the researchers would need to be sure that it is appropriate for a given level pairing before implementing it. The development of a procedure to determine if the proportional odds assumption is appropriate under the adjacent-categories logit link function would allow for its incorporation into the proposed model. In addition, the proposed model could be extended through the use of other link functions. For example, the cumulative logit link might be of interest for a given application and could be used if the proportional odds assumption was determined to be appropriate for that structure. An extension allowing for random intervention effects could also improve the fit of the proposed method for many networks.

## Conclusions

In conclusion, we have proposed a multinomial NMA model for ordinal outcomes that can simultaneously handle multiple outcome categorizations, thereby ensuring that data from all of the trials included in a network can be used during estimation. The use of the adjacent-categories logit link incorporates the ordering of the outcome into the comparative effect parameters, and simulations showed that the model generally performs well with respect to estimation. The inclusion of the general form of the modified multinomial likelihood that allows for any combination of levels and R functions linked to below that can be used to implement the method should allow for its use in a wide range of applications. Moreover, there is substantial room for further development that can take fuller advantage of the ordinality of the outcome through the proportional odds assumption and the utilization of additional link functions.

## Data Availability

The dataset analyzed in the application section of this article as well as R functions that can be used to implement the proposed method are available at https://github.com/psmorris15/ordinal_NMA.
